# Modified gastro-soleus turn-down flap for chronic or neglected achilles tendon ruptures

**DOI:** 10.1186/s13018-024-04625-y

**Published:** 2024-03-06

**Authors:** Mohamed A. A. Ibrahim, Mohamed G. Abdelkader, Samir A. Nematallah, Gamal A. Elsawy, Sameh A. Alghandour, Lotfy M. Shwitter

**Affiliations:** Al Azhar University-Faculty of Medicine-Orthopedic department., Cairo, Egypt

**Keywords:** Achilles' tendon, Chronic rupture, Gastro-zealous, Reconstruction, Turn down flap

## Abstract

**Background:**

Achilles’ tendon chronic rupture is a common entity that is usually misdiagnosed or mistreated. Hence, she was presented to us later or with complications affecting her gait. Surgical resection is needed to either bridge the gap or reinforce the strength of the tendon repair.

**Objectives:**

Our study's goal was to assess the clinical results of repairing chronic Achilles’ tendon lesions employing the middle segment of the proximal portion of the tendon (gastro-soleus), as a turn-down flap.

**Methods:**

Our prospective interventional single arm study included 18 patients with chronic Achilles’ tendon rupture attending at Al-Azhar university hospitals in Cairo, Egypt from May 2020 to April 2023. Diagnosis of the patients was confirmed by radiographic and clinical investigations. They were all treated with the same open reconstruction procedure using a modified GSF. The average follow-up was 12 months. The results of this study were assessed by the Achilles tendon rupture score (ATRS), American Orthopedic Foot and Ankle Society (AOFAS) score, and capacity to perform repeated heel raises on the affected side.

**Results:**

The mean operative time was 72.77 min. The median (IQR) time of reconstruction was 10 (8–12) after the injury. The median (IQR) length of flab was 4.5 (4.3–5) 9 (Table 2). No intraoperative complications occurred. The typical follow-up period was 12 months (6–18 months). In terms of the ATRS, we found a significant reduction from 82.8 ± 3 preoperatively to 20.8 ± 6.7 at 12 months postoperatively (*P* value = 0.001). As regards the AOFAS score, it was increased from 49.5 ± 10 preoperatively to 83.8 ± 8.5 12 months postoperatively (*P* = 0.001). In terms of the post operative complications, there was no re-rupture. Two patients experienced superficial wound infection which improved with daily dressing and antibiotics. Additionally, two patients had slight ankle stiffness four months after the operation, which improved after programmed rehabilitation at the sixth month.

**Conclusion:**

The modified GSTF is a simple, safe, well-tolerated and effective method of treatment with excellent functional results and greater patient content.

## Introduction

Achilles’ tendon rupture (ATR) is a somewhat prevalent injury that occurs most frequently in young to middle-aged, active adults between the ages of 30 and 50. In the UK, there is evidence of a second peak occurrence after age 60, but there are still 4500 registered ruptures annually, and there are more reports of such a pattern in other countries [1–3].

Sedentary lifestyle, smokers, Diabetes mellitus, hypercholesterolemia, thyroid disorders, and obesity, corticosteroid local infiltrations or had previous local tendonitis can impair the health of tendons, leading to Achilles tendinopathy and possibly predisposing patients to Achilles tendon ruptures [4, 5]. Typically, they are the individuals which have less plantar flexibility, agitation, trouble walking, and chronic discomfort [5, 6].

In most situations, acute Achilles tendon ruptures are easily diagnosed and treated. However, up to 25% of ATRs are overlooked, either because of a physician's incorrect diagnosis or because of the patient's incorrect interpretation of the injury and failure to seek immediate medical attention. Evaluation is more challenging and could result in a false diagnosis, which is typically an ankle sprain or ruptured calf muscle, when the patient's history is atypical and there has not been substantial traumatic [5, 6].

The injury may be considered chronic or ignored if the original tendon rupture is not readily recognized, as in up to 25% of the instances. Even though authors vary on the precise definition of the term "chronic" A systematic review by Flint et.al (10), Suggested that this term should be applied to characterize a rupture that manifests four weeks or more following the first injury [7], Nicola Maffulli, describes chronic ruptures of Achilles tendons as those that present four to six weeks after the initial injury [8].

A wide range of procedures, such as V–Y lengthening, transfer of the flexor hallucis longus tendon and free tendon grafts (semitendinosus autograft) have been described for treating chronic Achilles ruptures. There has been an increase in publications describing the use of various strategies for treating chronic Achilles ruptures [9–12].

An updated overview of the literature showed that there is no agreement on the best management strategies for the chronic ruptures of the Achilles tendon. Therefore, the objective of this research was to assess the clinical results of reconstructing chronic Achilles tendon defects with a modified turn-down GSF.

## Patient and methods

Our prospective interventional single arm study included 18 patients with chronic Achilles’ tendon rupture attending at Al-Azhar university hospitals in Cairo, Egypt from May 2020 to April 2023. Our study followed the Helsinki declaration principals, and ethical approval was obtained from our institution before the beginning of the study. Written informed consent was obtained from every patient at the time of recruitment. We included the patients according to the following criteria;

The inclusion criteria were patients who had a gap larger than 2 cm and were symptomatic with chronic rupture of the Achilles tendon four weeks or more following the injury.

The exclusion criteria were:

1. Previous operations for tendon repair
2. Patients with a history of open ruptures of the Achilles tendon
3. Concomitant ruptures with fractures
4. Gap defects less than 2 cm
5. Injuries less than 4 weeks
6. Poor skin conditions

### Data collection

Complete medical history and general examination were obtained from every patient at the time of recruitment. All patients underwent clinical evaluation for range of motion (passive dorsiflexion increase and passive plantar flexion decrease), tendon defects, and skin conditions. Neurovascular status and associated injuries. The Thompson test [13] was performed for all patients and the results were negative, which indicated that the squeezing of calf muscles did not show passive plantar flexion of the foot as normally occurs on the contra lateral healthy side.

The Matles test [14] was also performed and showed positive results compared with those of the normal side, which indicated no planter flexion of the foot in the prone position with 90° of knee flexion. The single heel lift test could not be completed by any of the patients.

**Fig. 1:**
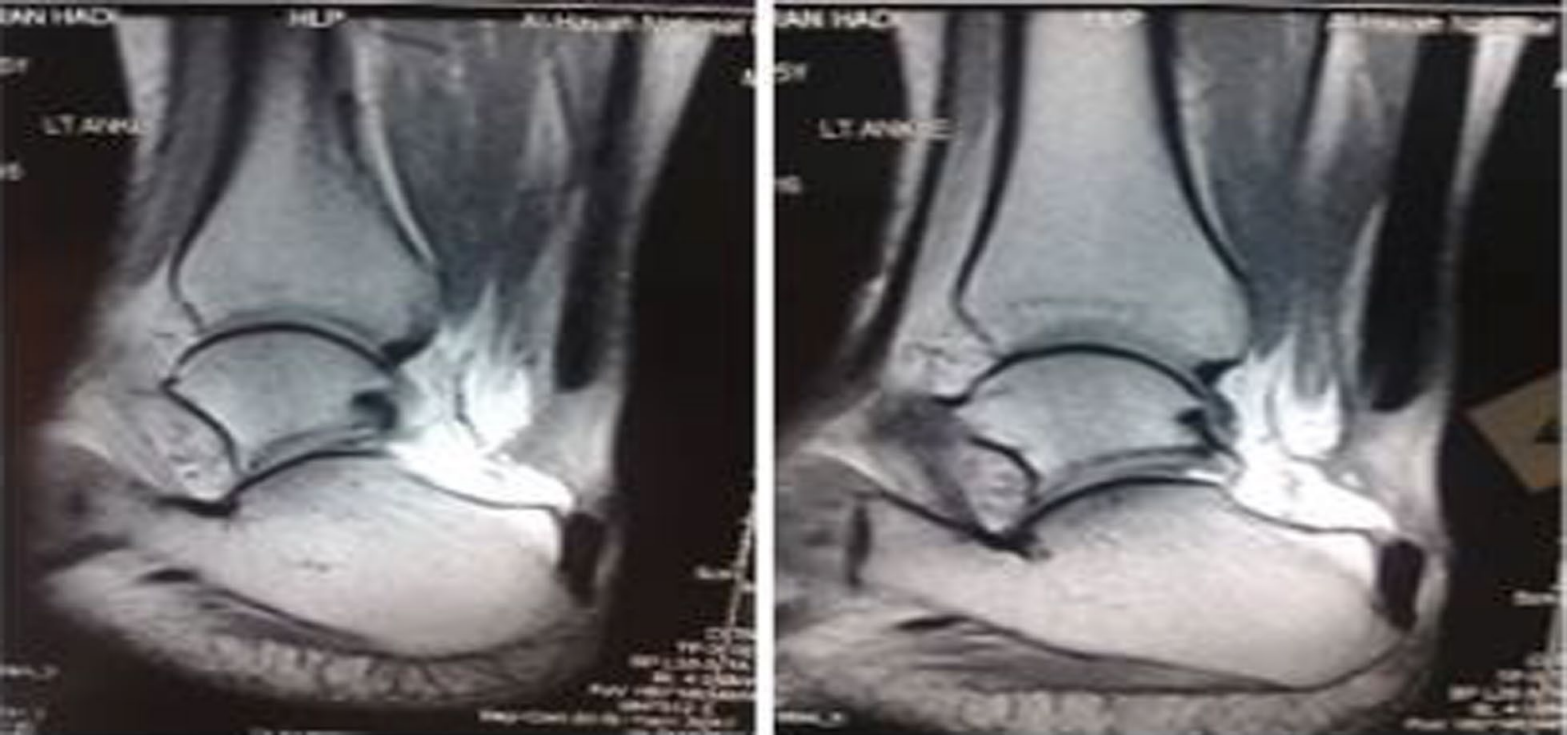
MRI image showing a gap greater than 2 cm in the torn Achilles tendon

Routine leg and ankle X-ray was used to exclude associated bone injuries, and ultrasonography and magnetic resonance imaging (MRI) were performed to confirm the diagnosis and measure the defect (Fig. 1).

**Fig. 2:**
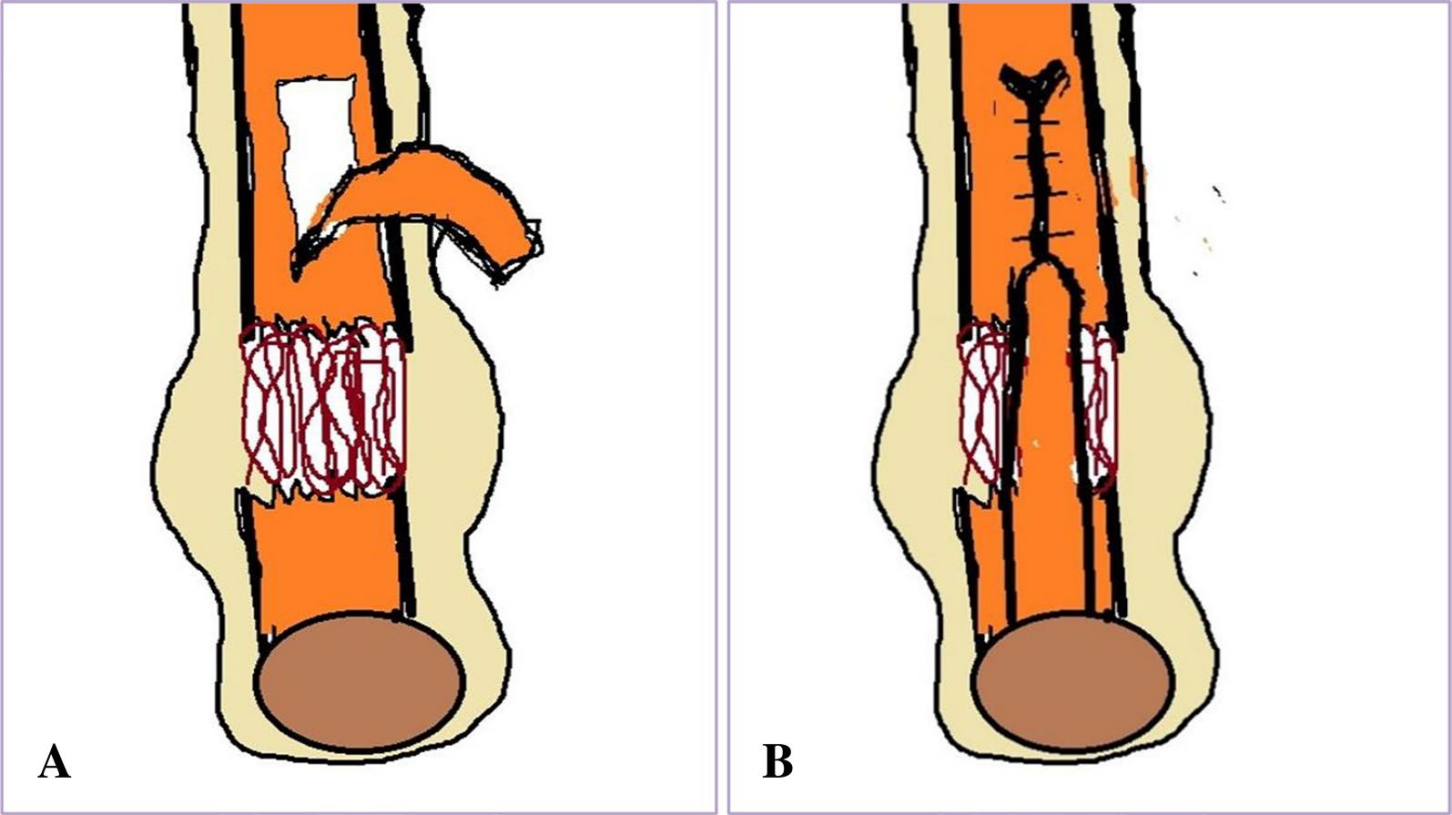
**A** Diagrammatic illustration of Turndown flap from proximal stump leaving its distal end intact. **B** Rotation of flap 180° on itself and suturing it to the distal stump

A modified turn-down flap of the gastrocnemius soleus was used in open surgery on these patients (Fig. 2). The American Orthopaedic Foot and Ankle Society (AOFAS) score and the Achilles tendon rupture score (ATRS) were utilized by the authors to assess the pattern of damage and ascertain the range of range of motion, assess comorbidities, and evaluate functional outcomes and patient satisfaction [14, 15]^.^

### Surgical technique

Prior to surgery, all patients underwent regular laboratory testing and cardiac, chest, and anaesthesia consultations to ensure that they were medically fit for the procedure. Consent was also obtained.

**Fig. 3:**
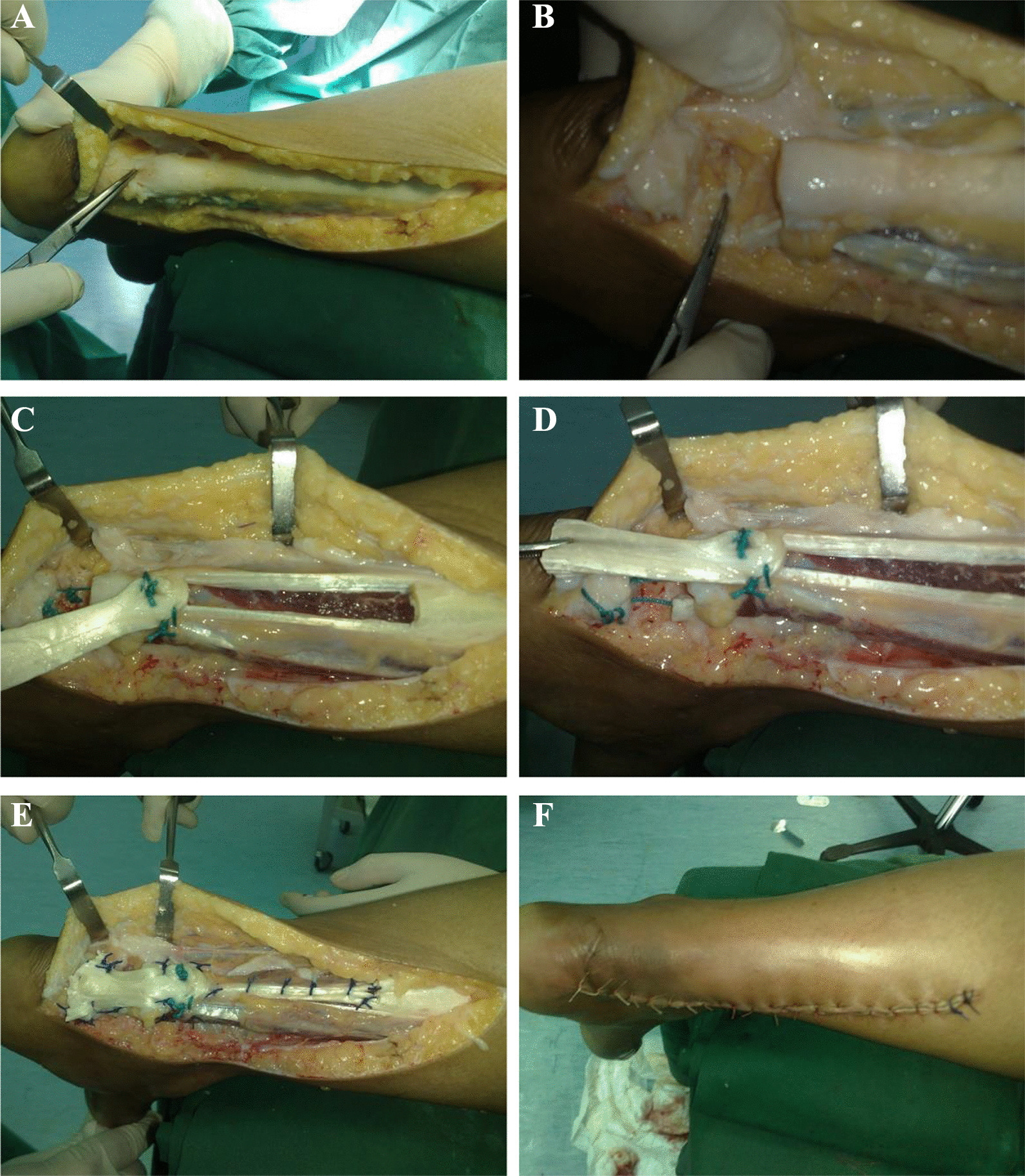
**A** Achilles tendon debridement. **B** Removal of degenerated tissue present between torn tendons end & measure the defect length **C** Turndown flap from the proximal stump leaving its distal end intact. **D** Rotation of flap 180° on itself. **E** Suturing of flap graft into the distal intact tendon stump and proximal into gastrocnemius defect **F** closure of the wound

All the patients included in the present study underwent identical surgical procedures. Using a thigh tourniquet and spinal anesthesia, all patients received prophylactic antibiotics during the induction of anesthesia. To facilitate easy movement and manipulation during surgery, all patients were placed prone, with the ipsilateral foot free and dangling at the end of the operating table. A skin incision was made over the posterior part of the lower leg, and the incision diverged medial to the foot to reach the ruptured tendon. Then, dissection was carried out in the subcutaneous tissue, fascia, and then the tendon sheath. After that, we remove the scar and degenerative tissue between the ruptured ends of the tendon (Fig. 3A), trimming the ends of the Achilles tendon during the ankle in plantar flexion up to 30°, and then assess the length of the defect (Fig. 3B). A turndown flap was taken from the proximal stump, leaving its distal end with contact to the proximal stump. The flaps were separated from the underlying muscle bellies and then rotated 180° on themselves rather than downward to preserve the gliding surface as it is, so that the same surface related to skin was still related to skin (Fig. 3C and D).

The flap was sutured to the proximal gastrocnemius defect and the distal Achilles tendon stump while the foot was mildly plantar flexed (Fig. 3E). Normal saline was used to irrigate the wound before closure (Fig. 3F).

### Postoperative Follow up

The ankle was immobilized in a 20-degree plantar flexion cast for 2–3 weeks and then in a neutral cast for 2–3 weeks. All participants were allowed to bear a protected weight after 8 weeks, and they were allowed to bear a full weight after 12 weeks. Participants underwent range-of-motion physiotherapy after the cast was taken off. Sports involving running and jumping are prohibited for 9–12 months, while riding and swimming are allowed after 12–16 weeks’ post-surgery. In the outpatient clinic, all patients underwent clinical follow-up, and any complications were noted.

### Statistical analysis

It was conducted using SPSS version 26 (Chicago, IL, USA). Normality of the data was tested by the Shapiro wilk test. Continuous variables were shown as the mean ± SD or median and interquartile range. Categorical variables were described as Numbers and percentages. Paired data were compared by using the paired *t*-test.

## Results

A total number of 18 patients diagnosed with chronic symptomatic rupture of the Achilles tendon 4 weeks after injury with a gap greater than 2 cm and were treated with open reconstruction via a modified gastrocnemius soleus turn-down flap were included in this study. The mean age of the studied patients was 40 ± 8.3 years. Thirteen patients were male (72.2%) and five patients were females (27.8%). According to the mechanism of injury, the most common cause was spontaneous (44.4%) following mild trauma during recreational sports or regular daily activity and had not responded to conservative treatment; no patients were impacted while participating in sports activities, followed by Misdiagnosed in 6 (33.3%) patients after presenting to the emergency room with complaints of posterior ankle pain and Neglected in 4 (22.2%) patients due to their choice of conservative measures for treating this injury. History of previous tendinitis was found only in eight patients (44.4%). As regards the time to surgery, the mean time was 11 ± 2.5 weeks (Table 1).

**Table 1:** Demographic and clinical data of the studied patients

Variables	N (%)/Mean ± SD (N = 18)
*Age (Years)*	
Mean ± SD	40 ± 8.3
Range	26–50
*Gender*	
Male	13 (72.2%)
Female	5 (27.8%)
*Side*	
Right	13 (72.2%)
Left	5 (27.8%)
*Cause of rupture*	
Spontaneous	8 (44.4%)
Neglected	4 (22.2%)
Misdiagnosed	6 (33.3%)
*Associated comorbidities*	
DM	8 (44.4%)
None	10 (55.6%)
*Time to surgery (weeks)*	
Mean ± SD	11 ± 2.5 5
6 weeks	1 Patient
8 weeks	4 Patients
10 weeks	8 Patients
12 weeks	2 Patients
14 weeks	2 Patients
16 weeks	1 Patients

Percentages per total

In our study most of the patients were manual workers 14 (77.7%), and only four patients (22.2%) were participating in recreational sports. No one remembers that he was complaining of pain at the site of his Achilles tendon prior to rupture. Dominant lower limbs were affected in eight patients (44.5%) while 10 (55.5%) patients were non dominant side affected.

According to the operative data, the median and IQR length of flab was 4.5 (4.3–5) cm. The mean operative time was 72.77 ± 6.2 min (Table 2). No intraoperative complications occurred. The typical follow-up period was 12 months (6–18 months).

**Table 2:** Operative data of the studied patients

Variables	Median (IQR)/Mean ± SD
Length of flap (cm)	4.5 (4.3–5)
Operative time (Minutes)	72.7 ± 6.2

In terms of the ATRS, we found a significant reduction from 82.8 ± 3 preoperatively to 20.8 ± 6.7 at 12 months postoperatively (*P* value = 0.001). As regards the AOFAS score, it was increased from 49.5 ± 10 preoperatively to 83.8 ± 8.5 12 months postoperatively (*P* = 0.001) (Table 3).

**Table 3:** Functional scores of the studied patients

Variables	Preoperative	Postoperative	*P* value^a^
ATRS	82.8 ± 3	20.8 ± 6.7	0.001*
AOFAS	49.5 ± 10	83.8 ± 8.5	0.001*

^a^Paired *t* test

In terms of the post operative complications, there was no re-rupture. Two patients experienced superficial wound infection which improved with daily dressing and antibiotics. Additionally, two patients had slight ankle stiffness four months after the operation, which improved after programmed rehabilitation at the sixth month.

**Fig. 4:**
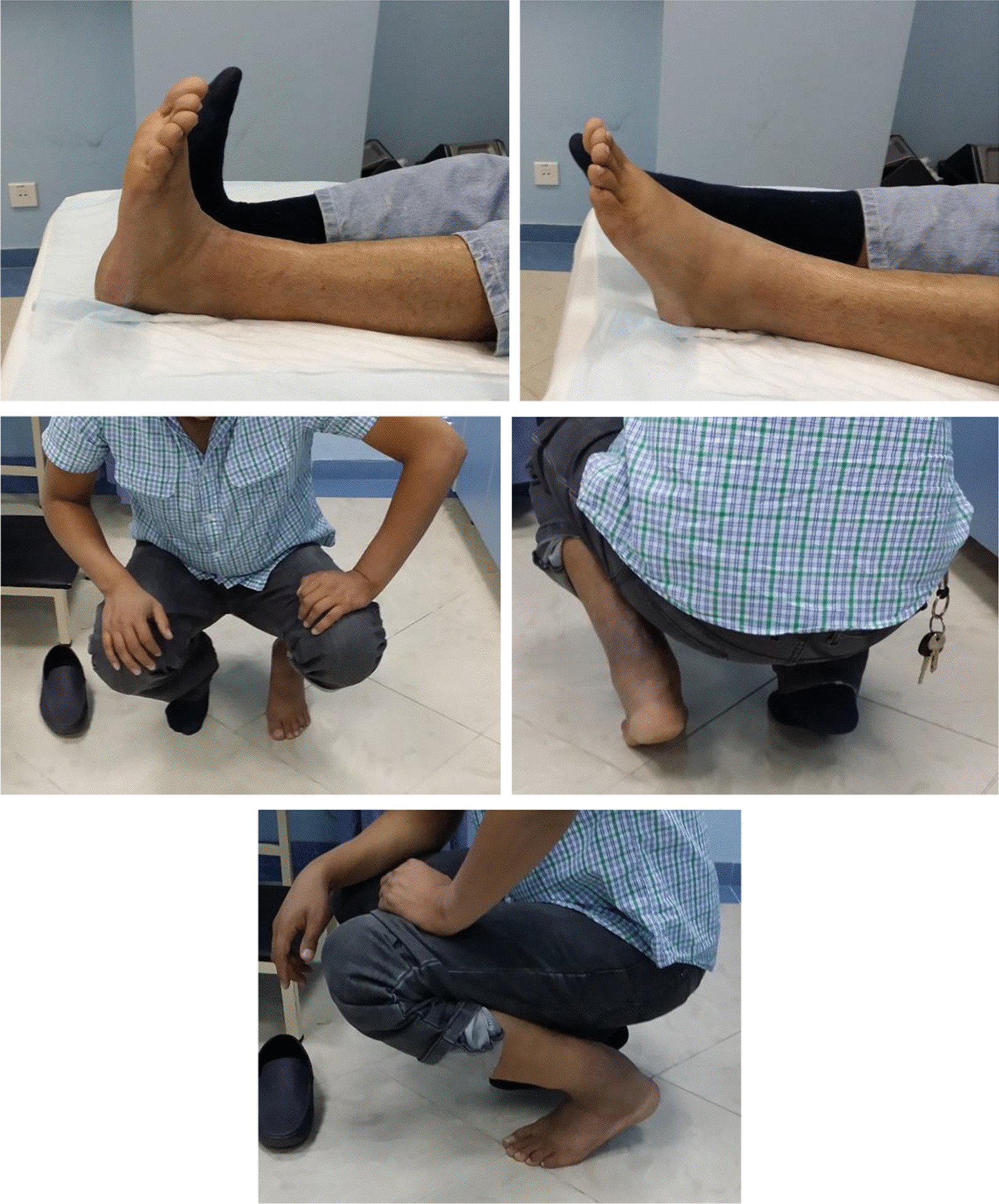
Functional results of the patient at 6 months after reconstruction

All patients experienced satisfactory outcomes as a result of the disappearance of disability, the recovery of ankle motion, and the improvement of physical activity without any significant complications (Fig. 4).

## Discussion

Achilles’ tendon ruptures typically happen 1–6 cm in front of the tendon's insertion into the calcaneal tuberosity. The Para-tenon frequently lacks tendinous tissue at the defect site, and dense scar tissue covers the area between the proximal and distal tendon stumps [15, 16]^.^

Achilles’ tendon contraction, decreased blood supply to the ruptured location, and gaps created by tendon degradation make treating chronic Achilles’ tendon rupture more challenging than treating acute rupture [15, 16] There is much controversy around the management of acute ruptures, and prior research has described both open surgical, percutaneous techniques like (Ma and Griffith) and (Tenolig) and conservative approaches [17–20] Studies indicate that non-operative care is associated with fewer time-consuming problems. The authors have demonstrated decreased re-rupture rates with open operational repair compared to non-operative techniques, making it the preferred alternative in the past [17–20].

For the reconstruction of chronic ATRs, a variety of surgical procedures have been described, such as the use of the V–Y tendinous flap, the transfer of tendon from the (peroneus brevis or flexor digitorum longus or flexor hallucis longus), the use of synthetic materials (Marlex mesh, polymers, carbon fiber, polyester, and a cellular dermal tissue matrix), the use of turndown flaps, free tendon auto-graft (hamstring) and the use of dual techniques [12, 21]. The extent of the defect is the only factor considered by many protocols for reconstructing chronic injuries of the Achilles tendon. Other parameters such as the age of the injury and the condition of the tendon ends have been taken into account in recent publications [22].

Chronic Achilles tendon injuries are categorized according to the extent of the defect by the Myerson protocol as follows; lesions with a defect less than 2 cm are treated with direct repair; A defect of 2–5 cm, is treated by V–Y advancement with sporadic tendon transfers and with augmentation when indicated; and a defect larger than 5 cm is treated primarily by tendon transfer in addition to V–Y lengthening if needed [23].

For reconstruction of a persistent Achilles tendon injury, a thorough review of conventional techniques was conducted. Two times after surgical repair, the rupture occurred again. In addition, there were further complications such as infection (7.6%), wound issues, such as scar hypersensitivity (4.7%), injury to the sural nerve (1.8%), deep vein thrombosis (DVT) (1.2%), post-operative tendonitis (1.2%) and discomfort [24].

In comparison to Yangjing Lin et al. [25], who employed a similar technique in 8 patients but with an inverted turndown flap, we obtained a similar outcome. All patients exhibited improvement in physical activity and reached their pre-injury activity level at final follow-up. As with our patients, they evaluated the patient’s postoperative activity using the AOFAS score and ATRS. The average AOFAS score was 60.13 preoperatively and 94.63 postoperatively, and an improvement of 34.5 points was obtained. Additionally, the average postoperative ATRS decreased from 92.62 preoperatively to 43.83 postoperatively with an average improvement of 48.79 points [25].

Zaki Arshad et al. [26] In a scoping review of 73 articles that met all the inclusion criteria, they found Complications in 50 studies involving 1063 patients and these complications were categorized as tendon re-rupture; infection (both superficial and deep); wound healing (hypertrophic scar, delayed wound healing, wound dehiscence, and/or gaping). The overall rate of complications was 15.8%, with infection being the most frequent event at 5.5% [26].

Lastly, in comparison to other procedures or techniques used for reconstruction mentioned in the literature, our modified gastro-soleus turn-down flap technique for treating chronic Achilles tendon ruptures offers several advantages. (1) Rapid intake, faster healing, and less adhesion through rotating the central part of the flap 180-degree upside down and not inverting it, which maintain the inner surface touching the inner structure and the outer surface touching the subcutaneous tissue. (2) Avoiding donor site morbidity which documented with tendon transfer and free tendon graft harvesting. (3) Biologic reconstruction which avoids the complications of synthetic graft includes the possibility of infection, an unfavorable tissue reaction, and the possibility that inert materials prevented this already devitalized area from healing.

One of the strengths of the present study is that we evaluated the patient’s physical activity after surgery using the same score used at another institution for chronic Achilles’ tendon rupture reconstruction (AOFAS and ATRS). As a result, comparing our outcomes with theirs is simple. This study has some limitations, one of which being the limited patient population. In addition, the study found that a smaller number of patients participated in the other trials that involved rebuilding chronic Achilles’ tendon rupture. Every research employs a distinct reconstruction technique. The application of several methodologies in a single study has been extensively documented in the literature.

## Conclusion

The Modified GST flap is a simple, safe, well-tolerated and effective method of treatment with excellent functional results and greater patient content.

## Data Availability

No datasets were generated or analysed during the current study.

## References

[1] Boyd RP, Dimock R, Solan MC, Porter E (2015). Achilles tendon rupture: how to avoid missing the diagnosis. Br J Gen Pract.

[2] Lemme NJ, Li NY, DeFroda SF, Kleiner J, Owens BD (2018). Epidemiology of achilles tendon ruptures in the united states: athletic and nonathletic injuries from 2012 to 2016. Orthop J Sports Med.

[3] Ganestam A, Kallemose T, Troelsen A, Barfod KW (2016). Increasing incidence of acute Achilles tendon rupture and a noticeable decline in surgical treatment from 1994 to 2013. A nationwide registry study of 33,160 patients. Knee Surg Sports Traumatol Arthrosc.

[4] Oliva F, Marsilio E, Asparago G, Giai Via A, Biz C, Padulo J (2022). Achilles tendon rupture and dysmetabolic diseases: a multicentric, epidemiologic study. J Clin Med.

[5] Taglialavoro G, Biz C, Mastrangelo G, Aldegheri R (2011). The repair of the Achilles tendon rupture: comparison of two percutaneous techniques. Strategies Trauma Limb Reconstr.

[6] Maffulli N, Via AG, Oliva F (2017). Chronic achilles tendon rupture. Open Orthop J.

[7] Flint JH, Wade AM, Giuliani J, Rue JP (2014). Defining the terms acute and chronic in orthopaedic sports injuries: a systematic review. Am J Sports Med.

[8] Maffulli N, Ajis A (2008). Management of chronic ruptures of the Achilles tendon. J Bone Joint Surg Am.

[9] Hadi M, Young J, Cooper L, Costa M, Maffulli N (2013). Surgical management of chronic ruptures of the Achilles tendon remains unclear: a systematic review of the management options. Br Med Bull.

[10] Lin Y, Yang L, Yin L, Duan X (2016). Surgical strategy for the chronic achilles tendon rupture. Biomed Res Int.

[11] Maffulli N, Bartoli A, Sammaria G, Migliorini F, Karlsson J, Oliva F (2023). Free tendon grafts for surgical management of chronic tears of the main body of the Achilles tendon: a systematic review. Knee Surg Sports Traumatol Arthrosc.

[12] Maffulli N, Del Buono A, Spiezia F, Maffulli GD, Longo UG, Denaro V (2013). Less-invasive semitendinosus tendon graft augmentation for the reconstruction of chronic tears of the Achilles tendon. Am J Sports Med.

[13] Thompson TC (1962). A test for rupture of the tendo achillis. Acta Orthop Scand..

[14] Matles AL (1975). Rupture of the tendo achilles: another diagnostic sign. Bull Hosp Joint Dis.

[15] Höher J, Livesay GA, Ma CB, Withrow JD, Fu FH, Woo SL (1999). Hamstring graft motion in the femoral bone tunnel when using titanium button/polyester tape fixation. Knee Surg Sports Traumatol Arthrosc.

[16] Malagelada F, Clark C, Dega R (2016). Management of chronic Achilles tendon ruptures-a review. Foot (Edinb).

[17] Deng S, Sun Z, Zhang C, Chen G, Li J (2017). Surgical treatment versus conservative management for acute Achilles tendon rupture: a systematic review and meta-analysis of randomized controlled trials. J Foot Ankle Surg..

[18] Yang X, Meng H, Quan Q, Peng J, Lu S, Wang A (2018). Management of acute Achilles tendon ruptures: a review. Bone Joint Res.

[19] Taglialavoro G, Biz C, Mastrangelo G, Aldegheri R (2011). The repair of the Achilles tendon rupture: comparison of two percutaneous techniques. Strategies Trauma Limb Reconstr.

[20] Ochen Y, Beks RB, van Heijl M, Hietbrink F, Leenen LPH, van der Velde D (2019). Operative treatment versus nonoperative treatment of Achilles tendon ruptures: systematic review and meta-analysis. BMJ.

[21] Nordenholm A, Senorski EH, Westin O, Nilsson Helander K, Möller M, Karlsson J (2022). Surgical treatment of chronic Achilles tendon rupture results in improved gait biomechanics. J Orthop Surg Res.

[22] Periasamy M, Venkatramani H, Shanmuganathan RS (2019). Management of chronic Achilles tendon injuries-review of current protocols and surgical options. Indian J Plast Surg.

[23] Myerson MS (1999). Achilles tendon ruptures. Instr Course Lect.

[24] Hadi M, Young J, Cooper L, Costa M, Maffulli N (2013). Surgical management of chronic ruptures of the Achilles tendon remains unclear: a systematic review of the management options. Br Med Bull.

[25] Lin Y, Yang L, Yin L, Duan X (2016). Surgical strategy for the chronic achilles tendon rupture. Biomed Res Int.

[26] Arshad Z, Lau EJS, Leow SH, Bhatia M (2021). Management of chronic Achilles ruptures: a scoping review. Int Orthop.

